# Parental Attachment, Adult-Child Romantic Attachment, and Marital Satisfaction: An Examination of Cultural Context in Taiwanese and Thai Heterosexual Couples

**DOI:** 10.3390/ijerph17030692

**Published:** 2020-01-21

**Authors:** Ching-Yu Huang, Skultip Sirikantraporn, Nipat Bock Pichayayothin, Julie M. Turner-Cobb

**Affiliations:** 1 School of Psychology, Keele University, Newcastle-Under-Lyme ST5 5BG, UK; c.s.huang@keele.ac.uk; 2 California School of Professional Psychology, Alliant International University, San Diego, CA 92131, USA; jill.siri@fulbright.edu.vn; 3 Fulbright University Vietnam, 105 Tôn Dật Tiên, Tân Phú, Quận 7, Hồ Chí Minh 700000, Vietnam; 4 The Faculty of Psychology, Chulalongkorn University, Rama 1 Rd, Pathumwan, Bangkok 10330, Thailand; nipat.p@chula.ac.th; 5 Department of Psychology, Bournemouth University, Poole BH12 5BB, UK

**Keywords:** romantic attachment, parental attachment, marital satisfaction, intergenerational, gender, cultural differences

## Abstract

Relationship dynamics between married couples can differ considerably, with varying impacts on relationship satisfaction. However, very limited research attention has been paid to how intergenerational attachment, relating to an individual’s perception of his/her own and that of his/her parents’ attachment, can affect marital dynamics within different cultural contexts. The current study examined associations between married heterosexual couples’ romantic attachment, perception of parental attachment, and marital satisfaction in 100 Thai couples (*M* age = 45.59 years, *SD* = 10.86) and 73 Taiwanese couples (*M* age = 39.55 years, *SD* = 9.13). Results revealed that romantic attachment anxiety was negatively associated with marital satisfaction in the Taiwanese couples; in the Thai couples, neither romantic attachment anxiety nor avoidance was associated with marital satisfaction. Husbands reported higher romantic attachment anxiety than their wives in Taiwan, but this was not observed in the Thai couples. Taiwanese wives reported higher scores on their perceived parental attachment avoidance than did their husbands; whereas the reverse trend was observed in the Thai couples. These findings highlight the need to consider intergenerational aspects of attachment in cultural contexts, and they have important implications for practitioners working with couples from Asian cultural backgrounds.

## 1. Introduction

Marriage is an integral part of people’s social and emotional well-being. A satisfactory marital relationship contributes to one’s general well-being, whereas marital dissatisfaction is associated with negative physical and mental health outcomes [1]. While healthy marriages provide a positive psychological and physical environment for both partners and their children to grow and thrive, marriages high in discord can carry health hazards by creating a high-stress environment, which is strongly associated with poorer health outcomes for the couple and their children [1]. A greater understanding of which factors may increase the chance of marital harmony is of much importance for individual social and emotional well-being.

Several factors can contribute to relationship quality and marital satisfaction, most notably the factor of attachment. According to attachment theory, individual differences in reactions to relational distress manifest in two underlying dimensions: attachment anxiety and attachment avoidance [2]. An anxious attachment is characterized by fear of rejection and abandonment and strong cravings for closeness, whereas individuals with avoidant attachment style often feel uncomfortable with closeness and have a strong desire to maintain emotional distance. Securely attached individuals possess attributes that foster close intimate relationships, resulting in both higher relationship satisfaction and quality [3]. By contrast, adults with insecure attachment styles (i.e., having high scores on attachment anxiety and/or attachment avoidance) have a strong dependence and unhealthy desire and demand for intimacy that may push partners away and result in higher negative relationship characteristics and lower levels of relationship satisfaction [4]. Typological approaches in adult romantic attachment research postulated four types of attachment. The common types of adult attachment proposed by Bartholomew and Horowitz [5] were (a) secure (positive model of self and of other, and low on both avoidance and dependence); (b) dismissing (positive model of self, negative model of other, high avoidance, and low dependence); (c) preoccupied (negative model of self, positive model of other, low avoidance, and high dependence); and (d) fearful (negative model of self and of other, high on both avoidance and dependence).

The association between early attachment experiences and attachment style in romantic relationships is well-established (see [6] for review). However, the underlying mechanisms involved in this process of intergenerational transmission of attachment on subsequent relationship experiences, and their reach in terms of extent and quality of relationships, remain unclear. Past research has mainly focused on the comparison between offspring of divorced families and those of intact families. For instance, parental marital discord [7], conflict, and resentment between the parents [8], as perceived by offspring, has been found to adversely affect the offspring’s own marital relationship. Compared to adults from families of divorced parents, adults from intact families reported more secure romantic attachment and higher trust and relationship satisfaction [9]. Furthermore, individuals from families of divorced parents were more likely to endorse negative beliefs about romantic relationships, learnt from their families of origin, whereas individuals from intact families were more likely to report that their families of origin showed them that marriage is enduring and that external pressures play an important role in relationships [8]. Such comparisons have frequently been based on the structural domain of divorced versus intact marriages rather than an examination of the underlying psychosocial mechanisms and characteristics of perceived parental attachment which may recapitulate within the next generation. In a US sample of predominantly Caucasian newlyweds, one study specifically examined parent–child attachment and romantic attachment in adulthood as intergenerational mediating variables between parental marital satisfaction and the adult child’s own marital satisfaction [10]. Partial mediation effects were found for both types of attachment as potential mechanisms of marital satisfaction, but gender effects were also observed whereby husbands’ but not wives’ marital satisfaction was mediated by anxious attachment styles [10].

However, the precise nature of these mechanisms of intergenerational transmission of attachment styles remain unclear. One of the key aims of the present study is to further clarify the relationship between offspring perception of parental marital attachment (regardless of the marital status of the parents) and the individual’s own marital attachment and impact of this attachment on their marital satisfaction.

### 1.1. Cross-Cultural Perspectives on Adult Romantic Attachment

Cultural beliefs might help explain the different patterns of adult attachment, yet cross-cultural research in this area has been limited. In a cross-cultural study of adult romantic attachment across 62 cultural regions, differences have been reported in prevalence of preoccupied attachment style in Southeast Asians (in Malaysia, Indonesia, and the Philippines) and East Asians (in Hong Kong, Taiwan, South Korea, and Japan) [11]. Using a measure and scoring criteria based on Western cultures, secure attachment was a normative form of adult romantic attachment in most (79%), but not in all, the cultures examined [11]. More specifically, attachment behaviors viewed as insecure avoidance in Western cultures appear to be normative in Taiwanese culture, and the development trajectories of romantic relationship and dating behaviors may be different in Chinese cultures (for example, see Li et al. [12]). Wang and Mallinckrodt [13] report Taiwanese participants as scoring higher on insecure anxiety items than their American counterparts. They found that most Taiwanese believed it was egocentric to tell their partner how they feel, because restraint in self-expression is a core value in Taiwanese culture, reflecting interpersonal respect, even between romantic partners; in contrast, American romantic partners viewed expressing feelings to their partner for comfort, as a healthy relationship behavior [13]. 

To our knowledge, there are only two published studies investigating romantic attachment in Thai culture. These report on the development of the adult attachment measure ECR-R [14] and gender differences in romantic attachment [15]; however, these two studies leave the question of to what extent Thai and Taiwanese share characteristics pertaining to adult romantic relationships unaddressed.

For psychological research, which is heavily Euro-American based, Taiwanese and Thai culture would typically be grouped together as “Asian” culture. Indeed, both Taiwanese and Thai cultures share relatively comparable levels of collectivism [16]. People in collectivistic cultures value social harmony and interdependency within their group memberships. Important life goals and decisions of a person tend to be interconnected with his/her relevant others [17,18]. Couples in these cultures have been found to be more likely to hold back their preferences to maintain harmony in romantic relationships and to be less likely to voice different opinions with their partners, to avoid confrontation [19].

However, Taiwanese and Thai cultures also possess very different sociocultural characteristics. Taiwanese and Thai cultures are divided into different cultural regions: Thais were classified as Southeast Asians, whereas Taiwanese were classified as East Asians. In Taiwan, and many other East Asian cultures, Confucianism is the dominant cultural doctrine, which affects many aspects of people’s family and marital relationships [20]. For instance, Conficianism emphasizes children’s obedience and compliance to parents, affecting the intergenerational interaction dynamics, with parents possessing authority over children [21]. Marriage is traditionally conceptualised as the continuation of the husband’s family linage rather than as the beginning of a new family [22]. In Thailand, the prevalence of Buddhism has substantially influenced many different aspects of Thai people’s lifestyles, values, and attitudes, including their family relationships [23]. The Buddhist emphasis on being content and satisfied with what one has may increase people’s sense of satisfaction in general. Given the similarities and differences between these two cultures in the sociocultural contexts underlining their family relationships, the current study attempts to achieve a more nuanced understanding of these two Asian cultures, as applied to the context of parental attachment and marital satisfaction and mechanisms underlying intergenerational transmission of romantic attachment and marital satisfaction.

### 1.2. Gender in Adult Romantic Relationship

Del Giudice’s [24] meta-analysis on gender difference in adult attachment demonstrated that, across cultures, men were more likely to score higher on avoidance and lower on anxiety compared to women, although the effect sizes among subsamples and cultural regions were small (Ds = 0.07 to 0.34). Moreover, gender differences were more evident in particular social contexts where insecure attachment was prevalent [25]. For instance, [26] found that male Chinese participants reported lower insecure avoidance but similar anxiety scores compared to female participants. However, a more recent meta-analysis of adult attachment in Chinese college students did not find gender differences in avoidance and anxiety scores [27]. In Thailand, men were more likely to report insecure attachment than women, reporting higher anxiety and avoidance scores [15]. Moreover, fearful attachment style (i.e., higher avoidance and anxiety scores) was more prevalent in male college participants, and preoccupied style (i.e., lower avoidance and higher anxiety) was more prevalent in female college participants [14]. Overall, these findings of the Thai samples and the Chinese sample were not in line with the meta-analysis results from [24], indicating that Thai and Chinese did not share the same patterns of gender differences in adult attachment, underscoring the importance of conducting cross-cultural research.

Some evidence also suggested gender differences in the associations between attachment and relationship satisfaction. In [28], a study with a sample of 354 American heterosexual couples, the majority of whom were Caucasian, with 17% Black and 3% Hispanic and Asian backgrounds, women high in attachment anxiety experienced lower levels of relationship satisfaction; meanwhile, men high in attachment avoidance tended to report their relationships more negatively and indicate lower levels of commitment. Another study with 305 American couples suggested women were less satisfied with their male partners who had higher attachment avoidance, whereas men were less satisfied with their female partners who had higher attachment anxiety [29]. However, the extent to which these findings would be replicated in different cultural groups still awaits verification.

### 1.3. The Current Study

The aim of the current study was to examine intergenerational transmission of marital satisfaction in relation to couple romantic attachment and perception of parental attachment, in the context of cultural and gender differences between Taiwanese and Thai married heterosexual couples. The current study focused on identifying cultural differences in two dimensions of adult romantic attachment, not on group membership within subtypes of adult attachment; therefore, the scores of attachment anxiety and attachment avoidance were used in our subsequent analyses. The following was hypothesized: (1) perceived parental attachment (anxious and avoidant) would be positively related to couples’ romantic attachment (anxious and avoidant, respectively) in both Thai and Taiwanese groups; (2) couples’ romantic attachment would be negatively associated with marital satisfaction in both Thai and Taiwanese groups; (3) there would be significant cultural and gender differences between the Thai and Taiwanese in their perception of parental attachment, couples’ romantic attachment, and marital satisfaction (specifically, Taiwanese couples were expected to report higher attachment anxiety and avoidance, as well as lower relationship satisfaction than Thai couples); and (4) couples’ romantic attachment would significantly account for their marital satisfaction, even after the effect of their age, gender, income, and cultural groups was controlled for.

## 2. Materials and Methods

### 2.1. Participants

One hundred and seventy-three married heterosexual couples participated in this study (73 couples from Taiwan, and 100 couples from Thailand; total *N* = 346). For the Taiwanese couples (TW), the ages of the participants ranged from 25 to 64 years (wives: *Mean* = 38.63, *SD* = 9.23; husbands: *Mean* = 40.48, *SD* = 9.01). For the Thai couples (TH), the ages of the participants ranged from 23 to 69 years (wives: *Mean* = 46.47, *SD* = 11.22; husbands: *Mean* = 44.71, *SD* = 10.29). On average, the TW couples had been married for 10.58 years (*SD* = 6.50, range = 4 months to 38 years), whereas the TH couples had been married for an average of 17.63 years (*SD* = 11.34, range = 7 months to 49.4 years). The majority (85.6% of the TW and 83.5% of the TH couples) of the participants reported having two primary parental figures, and most of the participants indicated that their parents were married (95.9% of the TW and 91.4% of the TH couples). The majority (80.8% of the TW and 83% of the TH) of the couples had at least one child with the current spouse. Both the Taiwanese and Thai participants were from a well-educated, middle-class background (see Table 1). It should be noted that much higher percentages of the participants in this study had completed tertiary education than in the Taiwanese general population (44.19% [30]) or the Thai general population (36.29% [31]); therefore, the findings should be interpreted with caution.

### 2.2. Procedure

Participants in both Taiwan and Thailand were recruited from various institutions’ participant pools, as well as advertisements in the universities, social media, and online forums. Interested participants responded to the researchers by email or via online-messaging applications. The researchers then sent the link to online questionnaires (traditional Chinese version for the Taiwanese couples and Thai version for the Thai couples), together with their couple IDs, via email to the participants, for them to respond at their convenience. Couple IDs were created by the researchers by using the combinations of researchers’ initials, number of participants, and A or B for each spouse, for example, NA14A for the husband and NA14B for the wife. The couples with matched IDs were included in the current study. Participants provided written informed consent, using an electronic signature, prior to completing the online questionnaires, after which they were given a raffle opportunity to win a $30 gift card, as a token of appreciation. All participants submitted their responses electronically through Qualtrics, and the data was converted into a SPSS file for data analyses, using SPSS version 24. The study was conducted in accordance with the Declaration of Helsinki, and the study was approved by the Institutional Review Board of California School of Professional Psychology, Alliant International University (Project Identification Code 1603023816).

### 2.3. Measures

Demographic questionnaire: The demographic questionnaire was designed for the current study, to acquire the following information: gender, age, highest degree of education, employment status, length of marriage, number of primary parental figures (those the participant considered to be their parents), and whether or not their parental figures were married.

The Experiences in Close Relationships-Relationship Structures Questionnaire (ECR-RS; [2]). The ECR-RS is a self-report measure that assesses attachment patterns in a variety of close relationships. For the purposes of this study, the target was narrowed to focus upon adult romantic relationships. The ECR-RS consists of nine 7-point Likert-scaled items, anchoring from 1 (strongly disagree) to 7 (strongly agree). The avoidance score was computed by averaging items 1–6, while reversing items 1, 2, 3, and 4. Averaging items 7–9 yielded the anxiety score. The ECR-RS has been widely utilized to measure attachment among individuals in romantic relationships, demonstrating good psychometric properties [2]. The reliabilities (Cronbach’s α) from our sample were 0.89 for the anxiety scale (0.90 for TW and 0.86 for TH, respectively) and 0.80 for the avoidance scale (0.79 for TW and 0.80 for TH, respectively).

The Experiences in Close Relationships-Relationship Structures Questionnaire Modified (ECR-RSM). The ECR-RS was modified to assess the participants’ perception of their parental figures’ attachment, retrospectively. The ECR-RSM adapted the nine items from the ECR-RS, to ask participants to recall how their parental figures related to each other, thereby measuring their parental figures’ attachment styles. The reliabilities (Cronbach’s α) were 0.93 for the anxiety scale (0.95 for TW and 0.90 for TH, respectively), and 0.90 for the avoidance scale (0.87 for TW and 0.94 for TH, respectively).

The Revised Dyadic Adjustment Scale (RDAS; [32]) is a 14-item self-report questionnaire that measures three overarching categories to assess the way in which people adjust in their relationship: (1) *consensus*, comprising of the subcategories of decision-making, values, and affection. (2) *Satisfaction*, which assesses the subcategories of stability and conflict regulation. (3) *Cohesion,* which measures subcategories of activities and discussion. Each item in the RDAS asks the respondents to rate certain aspects of the relationship on a 5- or 6-point scale. For the purpose of the study, only satisfaction subscale was used for subsequent analyses. Overall, past studies indicated good psychometric qualities of RDAS and its suitability in both research and clinical evaluations [33]. The reliabilities (Cronbach’s α) of the RDAS satisfaction subscale were 0.85 for the Taiwanese sample and 0.76 for the Thai sample.

### 2.4. Statistical Analyses

In order to perform subsequent statistical analyses, the couples’ scores for each variable (attachment anxiety and avoidance; perceived parental attachment anxiety and avoidance; and marital satisfaction) were calculated by averaging the husband and wife scores, with both carrying the same weight. Preliminary correlations (Pearson’s *r* for numerical and Spearman’s *ρ* for ordinal variables) were used to examine associations between demographic variables (participant age, family income, educational level, and length of marital relationship) and the variables of interest (romantic attachment anxiety and avoidance, perceived parental attachment anxiety and avoidance, and marital satisfaction) in order to confirm inclusion as control variables. In the main inferential analysis, first, in order to examine how gender and cultural groups affect each attachment dimension and marital satisfaction, while partialling out the effects of age, income, and length of marriage, a two-way MANCOVA was conducted, followed by subsequent pairwise comparisons with Bonferroni corrections applied. Second, correlational analyses were conducted on romantic attachment, perceived parental attachment, and marital satisfaction within each cultural group, while partialling out the effects of age and family income. Hierarchical regressions were then used to determine whether variations in demographic characteristics, cultural group, and romantic attachment accounted for scores on marital satisfaction. In the hierarchical regression analyses, gender, age, family income, and length of marriage were entered first; cultural group (dummy coded, TH = 1, TW = 0) was entered in the second step; and, finally, romantic attachment anxiety and avoidance were entered in the third step. Perceived parental attachment anxiety and avoidance were not associated with marital satisfaction and were thus excluded from the hierarchical regressions.

## 3. Results

### 3.1. Preliminary Analyses

Participants’ age was associated with romantic attachment anxiety (Pearson’s *r* (338) = 0.113, *p* < 0.05) and avoidance (*r* (338) = −0.111, *p* < 0.05), as well as perceived parental attachment anxiety (*r* (338) = −0.126, *p* < 0.05) and avoidance (*r* (338) = −0.141, *p* < 0.01). Family income was associated with romantic attachment anxiety (Spearman’s *ρ* (346) = −0.132, *p* < 0.05) and avoidance (*ρ* (346) = −0.114, *p* < 0.05). The length of marital relationship was associated with romantic attachment avoidance (*r* (340) = −0.109, *p* < 0.05), as well as perceived parental attachment anxiety (*r* (340) = −0.156, *p* < 0.01) and avoidance (*r* (340) = −0.160, *p* < 0.01). Because of these significant correlations, participants’ age, income, and length of marriage were included in subsequent analyses when romantic and perceived parental attachment were the outcome variables. Educational level was only associated with romantic attachment anxiety (*ρ* (346) = −0.154, *p* < 0.01), and it covaried with family income (*ρ* (346) = 0.226, *p* < 0.01); therefore, it was excluded in subsequent analyses.

### 3.2. Gender and Cultural Differences

The two-way MANCOVA demonstrated a significant main effect of cultural group (*F*_(5,323)_ = 9.231, Pillai–Bartlett trace = 0.143, *p* < 0.001, η^2^ = 0.125), as well as a significant interaction effect of cultural group*gender (*F*_(5,323)_ = 3.158, Pillai–Bartlett trace = 0.047, *p* < 0.01, η^2^ = 0.047, achieved statistical power = 0.879). Subsequent univariate analyses of variance (ANCOVAs) revealed significant effects for family income (covariate) on romantic attachment anxiety (*F*_(1, 327)_ = 5.243, *p* < 0.05) and avoidance (*F*_(1, 327)_ = 4.752, *p* < 0.05), and significant univariate effects for cultural group on romantic attachment anxiety (*F*_(1, 327)_ = 5.987, *p* < 0.05), romantic attachment avoidance (*F*_(1, 327)_ = 15.831, *p* < 0.001), perceived parental attachment anxiety (*F*_(1, 327)_ = 26.889, *p* < 0.001), perceived parental attachment avoidance (*F*_(1, 327)_ = 15.891, *p* < 0.001), and marital satisfaction (*F*_(1, 327)_ = 12.191, *p* < 0.01). The ANCOVAs also revealed significant interaction effects for cultural groups*gender on romantic attachment anxiety (*F*_(1, 327)_ = 40.807, *p* < 0.05) and perceived parental attachment avoidance (*F*_(1, 327)_ = 4.823, *p* < 0.05). 

Subsequent pairwise comparisons revealed that the Taiwanese couples reported higher attachment anxiety and avoidance both for themselves (anxiety mean difference (MD) = 0.331, *p* < 0.05; avoidance MD = 0.569, *p* < 0.001), as well as their perceptions of their parents (anxiety MD = 0.875, *p* < 0.001; avoidance MD = 0.699, *p* < 0.01), than did the Thai couples. Additionally, the Thai couples reported higher marital satisfaction than did the Taiwanese couples (MD = 1.133, *p* < 0.01). The mean scores and standard deviations of each group and gender are summarized in Table 2. 

### 3.3. Associations between Romantic Attachment, Perceived Parental Attachment, and Marital Satisfaction

The partial correlational analyses (see Table 3) demonstrated similar patterns of correlation among romantic attachment, parental attachment, and marital satisfaction between the two groups. Romantic attachment anxiety was negatively correlated with marital satisfaction (*r* = −0.224, *p* < 0.01) in Taiwanese couples only, whereas romantic attachment avoidance was positively correlated with perceived parental attachment anxiety (*r* = 0.263, *p* < 0.01) only in Thai couples.

The hierarchical regressions results (see Table 4) revealed that, when the outcome variable was marital satisfaction, gender, age, family income, and length of marriage explained only 0.7% of the variance (*R*^2^ = 0.185, *F*_(4, 329)_ = 0.612, *p* = 0.654), and the model was not significant. The addition of cultural groups increased the proportion of variance explained (∆*F*_(1, 328)_ = 12.259, *p* < 0.001; ∆*R*^2^ = 0.036), and adding romantic attachment anxiety and avoidance also significantly increased the variance explained (∆*F*_(2, 326)_ = 3.657, *p* < 0.05; ∆*R*^2^ = 0.021).

The regression coefficients indicated that the married couples in Thailand reported significantly higher marital satisfaction than the couples in Taiwan, even when the effects of gender, age, income, and length of marriage were accounted for. Moreover, the regression coefficients also indicated that, even after the effect of gender, age, income, length of marriage, and cultural groups were accounted for, couples with higher attachment anxiety reported significantly lower levels of marital satisfaction.

In summary, these results revealed significant cultural group differences in all aspects of romantic attachment, perceived parental attachment, and marital satisfaction, while, generally, similar patterns of associations between romantic attachment and perceived parental attachment among these two cultural groups were observed. However, romantic attachment anxiety was negatively associated with marital satisfaction only in the Taiwanese couples, whereas, in the Thai couples, neither romantic attachment anxiety nor avoidance was associated with marital satisfaction. Moreover, husbands reported higher romantic attachment anxiety than their wives in Taiwan, but this was not observed in the Thai sample. Taiwanese wives reported higher scores on their perceived parental attachment avoidance than did their husbands, whereas the reverse trend was observed in the Thai couples. The results of the hierarchical regression analyses revealed that the cultural group significantly accounted for couples’ marital satisfaction; and romantic attachment anxiety significantly decreased the couples’ marital satisfaction, even after the other variables were accounted for.

## 4. Discussion

This study examined intergenerational transmission of marital satisfaction in relation to couple romantic attachment and perception of parental attachment, in the context of cultural and gender differences between Taiwanese and Thai married heterosexual couples. Our findings regarding intergenerational transmission of attachment were largely in line with Hypothesis 1 and past findings (for example, [6,10]). Perceived parental attachment avoidance was associated with romantic attachment anxiety and avoidance in Taiwanese, as well as in Thai, couples; and perceived parental attachment anxiety was associated with romantic attachment anxiety in both Taiwanese and Thai couples. However, perceived parental attachment anxiety was only correlated with romantic attachment avoidance in the Thai participants, not in the Taiwanese participants. The nonsignificant findings for the Taiwanese couples were also in line with the [26] study that found no connection between perceived quality of parental and adult attachment in the Chinese sample. Although perceived parental attachment may affect one’s interpersonal interaction model in a romantic relationship, other factors, such as experiences in familial and extra-familial relationships that are closer to the time of measurement, may have a stronger impact on the way individuals rate their romantic attachment. Potential factors, such as distress tolerance and cognitive-emotion-regulation strategies [34] may mediate the negative effects of maladaptive attachment styles. Direct and indirect experiences in relationships during adulthood cumulatively shape a person’s perspective of his/her attachment toward his/her romantic partner, albeit indirect experience may act in a subtler manner.

Our findings regarding associations between couples’ romantic attachment and marital satisfaction only partially supported Hypothesis 2, revealing a negative association between attachment anxiety (but not avoidance) and marital satisfaction for Thai and Taiwanese couples. The findings were not in line with past studies that indicated negative relationships between attachment anxiety, as well as avoidance, and marital satisfaction [3]. Finding suggest that attachment anxiety might universally explain marital dissatisfaction in Thai and Taiwanese, whereas attachment avoidance could not, and this has implications for extension to other cultures. The lack of association between attachment avoidance and marital satisfaction may be partially explained by cultural beliefs about marriage and ideal intimate relationships in Asian cultures. For instance, the items in the attachment avoidance measure represent the idea of open communication about problems and concerns with romantic partner, which is considered appropriate and constructive to intimate relationships in individualistic cultures. However, Thai and Taiwanese people may not view such behaviors as constructive, but instead as threats to their marriage, because self-expression in collectivist cultures is believed to disrupt their interpersonal harmony [13,19]. Therefore, when considering norms within Thai and Taiwanese cultures, having higher scores in attachment avoidance may have little to do with having attachment insecurity, but be more relevant to how intimate interpersonal interactions are conceptualized and operationalized in specific cultural contexts.

The significant cultural and gender differences in our findings were consistent with Hypothesis 3. Specifically, we found similar gender effects to those previously identified (for example, [10]), but this gender difference was also culturally specific. As expected, Taiwanese males reported having higher attachment anxiety than Taiwanese females, whereas such a gender difference was not significant in the Thai participants. This finding echoed findings from a previous study, using a Chinese sample [26], that revealed consistent gender difference patterns in romantic attachment anxiety among East Asian cultural samples, but not for the Thai sample, underscoring its different Buddhist Southeast Asian cultural context. Moreover, Taiwanese females reported having higher perceived parental attachment avoidance than did the Taiwanese male participants, whereas, in the Thai samples, the male reported higher scores than Thai females.

The differences in self-construal (i.e., orientation toward independence/dependence in romantic relationship) in relation to cultural norms may help explain the cultural and gender differences found in the current study [35]. The perceived expectations of sons in Chinese/Taiwanese culture to be the family leader, holding up family legacy and pride, may have explained the higher scores on anxious attachment in Taiwanese men [36]. The women’s movement has provided education, work, and political opportunities for Taiwanese women [37]. Modern Taiwanese women tend to be financially independent, prefer equality in romantic relationships, and feel less hesitant to leave an unhappy marriage. By contrast, most Taiwanese men still prefer to keep the traditional male-dominant role; therefore, they may feel anxious to maintain their romantic relationships to save face from being labeled as being a failure in marriage. The Taiwanese couples, similar to their Chinese peers, were less likely to have open communication with their romantic partner, leading to their reporting lower relationship satisfaction [38]. On the contrary, gender differences were not significant in the Thai sample, despite previous research [15] finding that Thai men were more likely to report higher anxiety and avoidance scores than women. Moreover, another study [14] with college participants revealed higher prevalence of fearful attachment style (i.e., higher avoidance and anxiety scores) in males and higher prevalence of preoccupied style (i.e., lower avoidance and higher anxiety) in females. It is possible that our findings did not replicate previous research, due to recent rapid modernization of Thailand and the higher educational levels in our sample than the Thai population. Future research with a more representative sample is needed to verify the findings regarding gender differences in romantic attachment.

Finally, we expected that romantic couples’ attachment anxiety and avoidance would significantly account for their marital satisfaction, even after the effect of their age, gender, income, and cultural groups were controlled for (Hypothesis 4). Substantial evidence has shown the positive link between attachment anxiety, avoidance, and relationship dissatisfaction [39]. Our findings only partially confirmed the previous research findings that attachment anxiety, but not attachment avoidance, contributed to couples’ marital satisfaction. The lack of association between attachment avoidance and marital satisfaction may be partially explained by cultural beliefs about marriage and the ideal intimate relationship in Asian cultures. By contrast, research findings with Spanish couples [40] revealed that attachment avoidance, but not anxiety, was associated with relationship dissatisfaction. These cultural differences highlighted the importance for future research to further understand the role cultures and cultural beliefs play, in order to better foster satisfying marital relationships.

### Limitations and Future Directions

The current study pioneered the examination of romantic attachment, as well as perceived parental attachment, in both Taiwanese and Thai married couples, providing valuable information on such relationships in the context of these under-researched groups. However, some limitations need to be acknowledged. The first, which was the major limitation, was that the interdependence data were treated as independent in our analyses, leading to the possibility of type II error [41]. The Actor–Partner Interdependence Model (APIM) would be the appropriate approach to dyadic data. However, our research goals of investigating cultural differences and intergenerational attachment required data sets with larger sample sizes, from both countries in order to conduct the two-member–two-group actor–partner interdependence mediated model (2M2G APIM [42]); we need to use SEM with perceived parental anxiety and avoidance attachment from husbands and wives as the exogenous variables, perceived romantic anxiety and avoidance attachment from husbands and wives as the mediators, and husbands’ and wives’ marital satisfaction as the outcome variables. The existing sample sizes were insufficient to estimate the parameters, considering the complexity of the proposed APIM. For this reason, we decided to conduct the analyses and interpret the findings with the cautions of the potential errors. Second, the study design (online questionnaires) and recruitment strategy (via participant pool and online recruitment) may have limited the sample variability, compromising the generalizability of the findings. Moreover, the Thai participants were slightly older than the Taiwanese couples. Our statistical analyses have controlled for the effect of participant age; thus, our findings are valid, despite the small age difference noted between the cultural groups. To verify our study findings, a replication study composed of a more representative and age-compatible sample is needed. Third, the reliance on self-report without other types of collateral information, compromised the validity of the data. The current study only used the adult-child reports of attachment styles, both for themselves and for their parents, and they are positively correlated. Thus, the shared variance between them made the interpretation of data difficult. Future studies containing observational data or various informants (such as collecting both child and parental reports) would be critical in verifying our current findings. Forth, similar to most attachment research, we assumed that attachment insecurities lead to relationship dissatisfaction, but it is also possible that marital dissatisfaction leads to couples’ attachment insecurity. However, we are unable to assert the direction of influence with the cross-sectional data. This needs to be further ascertained in future longitudinal studies. Finally, future research should examine the effects of potential mediators (such as cognitive emotion regulation strategies, conflict resolution strategies, and personality) between attachment and relationship satisfaction, to obtain a more comprehensive picture. 

## 5. Conclusions

These findings reveal romantic attachment anxiety to be negatively associated with marital satisfaction in Taiwanese couples, whereas, in Thai couples, neither romantic attachment anxiety nor avoidance was associated with marital satisfaction. Husbands in our sample reported higher romantic attachment anxiety than their wives in Taiwanese, but not in Thai, couples. Taiwanese wives reported higher scores on their perceived parental attachment avoidance than did their husbands, whereas the reverse trend was observed in the Thai couples. The current study furthers our understanding of romantic attachment, intergenerational transmission of attachment, and marital satisfaction in two understudied cultural groups. The findings highlight the unique and important role played by cultural contexts in contributing to our understanding of the perception of both self and parental attachment in marital relationships. It underscores the importance of intergenerational consideration and the need for researchers and practitioners to exercise a more nuanced understanding of the influence of cultural differences in relationships between attachment and marital satisfaction.

## Figures and Tables

**Table 1:** Educational level and employment status of the participants by country and gender.

Variables	Levels	Taiwanese		Thai	
		Wives	Husbands	Wives	Husbands
Education	Up to high school	3 (4.1%)	8 (11%)	8 (8%)	5 (5%)
	College equivalent	42 (58.9%)	36 (49.3%)	54 (54%)	61 (61%)
	Postgraduate	27 (40.3%)	29 (39.7%)	36 (36%)	32 (32%)
Employment	Employed for wages	43 (58.9%)	49 (67.1%)	49 (49%)	56 (56%)
	Self-employed	10 (13.7%)	18 (24.7%)	27 (27%)	22 (22%)
	Homemaker	15 (20.5%)	18 (24.7%)	2 (2%)	8 (8%)
	Retired	2 (2.7%)	3 (4.1%)	9 (9%)	4 (4%)
	Students	NA	NA	4 (4%)	3 (3%)

**Table 2:** Cultural group and gender differences in romantic attachment, perceived parental attachment, and marital satisfaction.

Variables	Samples	Taiwanese (TW)		Thai (TH)		All		Mean Difference
		Mean	SD	Mean	SD	Mean	SD	TW–TH ^a^
ECR-RS ^b^ anxiety	Wives	2.37	1.04	2.45	1.14	2.42	1.09	−0.08
	Hus-bands	2.76	1.11	2.28	1.11	2.48	1.14	0.48
	All	2.56	1.10	2.37	1.12	2.45	1.11	0.19
ECR-RS avoidance	Wives	2.74	1.31	1.98	1.28	2.32	1.34	0.76
	Hus-bands	2.66	1.24	2.21	1.32	2.40	1.30	0.45
	All	2.70	1.27	2.10	1.30	2.36	1.32	0.60
ECR-RSM ^c^ anxiety	Wives	4.09	1.57	3.18	1.51	3.58	1.59	0.91
	Hus-bands	3.99	1.41	3.05	1.31	3.44	1.43	0.94
	All	4.04	1.49	3.11	1.41	3.51	1.51	0.93
ECR-RSM avoidance	Wives	3.37	1.42	2.24	1.47	2.74	1.55	1.13
	Hus-bands	3.00	1.32	2.59	1.73	2.76	1.58	0.41
	All	3.19	1.38	2.42	1.61	2.75	1.56	0.77
Marital Satisfaction	Wives	14.48	3.17	15.86	2.59	15.25	2.93	−1.38
	Hus-bands	15.23	2.69	15.92	2.69	15.63	2.70	−0.69
	All	14.85	2.96	15.89	2.63	15.44	2.82	−1.04

^a^ The mean difference scores = the scores from Taiwanese sample - scores from Thai sample. ^b^ ECR-RS = scores of The Experiences in Close Relationships-Relationship Structures Questionnaire. ^c^ ECR-RSM = The Experiences in Close Relationships-Relationship Structures Questionnaire Modified, assessing the participants’ perception of their parental figures’ attachment.

**Table 3:** Partial correlations between romantic attachment, perceived parental attachment, and marital satisfaction in the two groups (controlling for the effect of gender, age, family income, and length of marriage).

Variables	1		2		3		4	
	TW	TH	TW	TH	TW	TH	TW	TH
1.ECR-RS anxiety	---	---						
2.ECR-RS avoidance	0.313 **	0.508 **	---	---				
3.ECR-RSM anxiety	0.169 *	0.350 **	0.075	0.263 **	---	---		
4.ECR-RSM avoidance	0.243 **	0.199 **	0.399 **	0.387 **	0.467 **	0.568 **	---	---
5.Marital Satisfaction	−0.224 **	−0.088	−0.055	−0.066	0.010	−0.087	0.003	−0.054

* *p* < 0.05, ** *p* < 0.01.

**Table 4:** Results of hierarchical multiple regressions accounting for marital satisfaction.

Variables	Marital Satisfaction					
	Model 1		Model 2		Model 3	
	B	β	B	β	B	β
Step 1						
Gender	−0.380	−0.067	−0.358	−0.063	−0.379	−0.067
Age	0.024	0.089	0.028	0.103	0.037	0.136
Family income	−0.086	−0.042	−0.058	−0.028	−0.093	−0.045
Length of marriage	−0.021	−0.085	−0.040	−0.158	−0.043	−0.169
Step 2						
TW vs. TH			1.136 **	0.199	1.028 **	0.180
Step 3						
ECR-RS anxiety					−0.384*	−0.152
ECR-RS avoidance					0.018	0.009
Model Summary	R^2^ = 0.007		Δ R^2^ = 0.036		Δ R^2^ = 0.021	
	*F* (4, 329) = 654 (not significant)		*F* Δ (1, 328) = 12.259 **		*F* Δ (2, 326) = 3.657 *	

* *p* < 0.05, ** *p* < 0.01.
